# Effects of dietary fermented passion fruit (*Passiflora edulis Sims*) peel on growth performance, intestinal morphology, and cecal microbiota in Lingshan broilers

**DOI:** 10.3389/fnut.2026.1792055

**Published:** 2026-04-16

**Authors:** Siwei Nong, Zhenglu Liu, Rong Wan, Zhaoxiong Wang, Yerong Wang, Kaijun Wang, Shuibao Shen

**Affiliations:** 1 College of Agriculture and Food Engineering, Baise University, Baise, China; 2 College of Subtropical Characteristics, Agricultural Industry, Baise University, Baise, China; 3 College of Animal Science and Technology, Yangtze University, Jingzhou, China; 4 Hunan Provincial Key Laboratory of the Traditional Chinese Medicine Agricultural Biogenomics, Changsha Medical University, Changsha, China; 5 Research Institute of Agricultural and Animal Husbandry Industry Development, Guangxi University, Nanning, China

**Keywords:** Cecal microbiota, growth performance, intestinal morphology, Lingshan broiler, passion fruit peel

## Abstract

Passion fruit peel is a common byproduct of industrial passion fruit processing, yet it serves as a valuable source of diverse bioactive compounds and nutrients. However, limited attention has been paid in the literature to the nutritional properties and practical applications of passion fruit peel. This study aimed to examine the effect of fermented passion fruit peel (FPFP) on growth performance, intestinal morphology, and cecal microbiota of Lingshan broilers. A total of 400 female Lingshan broilers were randomly allocated to four groups with five replicates per group and 20 birds per replicate. The control group (CK) was fed a basal diet, while the experimental groups had 5% (L5), 10% (L10), and 15% (L15) of the basal diet replaced with FPFP. Growth performance and intestinal morphology were determined, and the cecal microbial community was analyzed via 16S rRNA sequencing. The results demonstrated that the 10% inclusion yielded optimal outcomes. Compared to all other groups, the 10% group exhibited significantly higher final body weight, total weight gain, and average daily gain (*p <* 0.05), coupled with a significantly lower feed-to-gain ratio (*p <* 0.05), while feed intake remained unaffected. Morphologically, the 10% group showed significantly greater duodenal villus height and higher villus height-to-crypt depth ratio in the jejunum and ileum (*p <* 0.05). Total intestinal length was also significantly increased in all FPFP-supplemented groups (*p <* 0.05). Although overall cecal microbial community structure (beta diversity) was not significantly altered, beneficial taxonomic shifts were observed. Specifically, the relative abundances of fibre-degrading Rikenellaceae_RC9_gut_group and growth metabolism-associated *Oscillibacter* were significantly enriched in the 10 and 15% groups (*p <* 0.05). In conclusion, dietary inclusion of 10% FPFP significantly enhances broiler growth performance and feed efficiency. These benefits are likely mediated through improved intestinal absorptive architecture and a modulated cecal microbiota, supporting the potential of FPFP as a functional feed resource for sustainable poultry farming.

## 1 Introduction

Passion fruit (*Passiflora edulis Sims*), a perennial evergreen liana of the family Passifloraceae, is native to South America and is now widely cultivated in tropical and subtropical regions globally (1). In China, the primary production areas are Guangxi, Guangdong, Hainan, and Fujian, with major cultivated varieties including Tainong No. 1 (purple fruit), Golden (yellow fruit), and Merlin Star (purple-red fruit) (2). Renowned as the “king of fruit juices”, passion fruit possesses a rich aroma and distinctive flavour, and is abundant in bioactive compounds such as vitamins, amino acids, flavonoids, and polyphenols (3–8). In recent years, the rapid expansion of the passion fruit processing industry has generated substantial by-products. Passion fruit accounts for 52% of the processing residue within the juice business, and a whopping 85% of which is peel, with only 17% being seeds (4). Passion fruit peel is rich in many nutrients containing crude protein (6.47–7.50%), fibre (61.70%), total lipid (0.40–4.89%), carbohydrates (80.7%) and ash (7.93%) (5, 9).

However, this material is prone to mildew and spoilage, presenting significant difficulties in storage and utilisation. Currently, the majority of this by-product remains underutilised, leading to resource wastage and potential environmental pollution. Emerging research indicates dietary polysaccharides from yellow passion fruit peel may improve on the dextran sulfate sodium-induced colitis model in mice (10). It has been shown to enhance immune function and gut function in Sanhuang broilers by improving antioxidation and short-chain fatty acids and decreasing inflammatory cytokines (11). Passion fruit peel can also enhance the growth and cecal microbiota of Nile tilapia (12). The extracts of passion fruit peel have been shown hepatoprotective activity and nephroprotective activity with a dose dependent activity (13).

In recent years, fermentation treatment has been an effective tool for recycling agro-industrial residues in useful animal feeds. Fermentation treatment has improved the nutritional composition of sour cherry kernels (14). Currently, research into the gut microbiota of both animals and humans is advancing, unveiling metabolic function of the gut microbiota. Simultaneously, there is a continuous emergence of developments in gut microbiota preparations (15, 16). As sequencing technology continues to advance, we could delve deeper into the influence of dietary changes on the gut microbiome of animals (17–19). However, no studies have been reported on the effects of fermented passion fruit peel on the growth performance, intestinal morphology and cecal microbiota of broilers. Therefore, this study intends to subject passion fruit peel to microbial fermentation treatment and incorporate them at specific inclusion levels as a replacement for the basal diet of broilers. By systematically evaluating their impact on broiler growth performance, intestinal morphology and cecal microbiota, this research aims to provide data support and a theoretical basis for the scientific application of passion fruit by-products in broiler feed industry.

## 2 Materials and methods

The Baise University Animal Care and Use Committee reviewed and approved all protocols (the approved protocol number: BSUCKL20250014) used in this study.

### 2.1 Diets, experimental design and management

A total of four hundred forty healthy eighty-day-old female broilers (Guangxi Lingshan native chickens) were purchased from a commercial local hatchery and used in this experiment. At arrival, chickens were weighed and assigned to treatment groups so that the initial weight was similar among different treatment groups. Five replicate pens of 20 broilers were randomly allotted to each of 4 treatments based on a single-factor completely randomized design. The control group (with no supplemental fermented passion fruit peel, CK) was fed a basal diet formulated to meet nutrient requirements provided by the Chinese Feeding Standard for Chickens (Table 1). The experimental diets (designated L5, L10, and L15) had 5, 10, and 15% of the basal diet isocalorically and isonitrogenously replaced with FPFP (fermented passion fruit peel), respectively (20). A 7-day pre-trial period was followed by a 49-day formal trial, and the samples were collected into tubes using the methodology described in a prior study (21).

**Table 1:** Basal diet composition and nutritional level (dry matter basis).

Raw material	Proportion (%)	Nutrient	Content
Corn	60.00	Dry matter (%)	87.87
Soybean meal	20.00	Ether extract (%)	6.43
Calcium hydrogen phosphate	1.50	Crude fiber (%)	3.46
Fish meal	3.00	Starch (%)	43.93
Rice bran	6.00	Crude protein (%)	17.60
Wheat bran	5.00	Ash (%)	4.60
Stone powder	1.08	Calcium (%)	0.67
Premix^a^	1.00	Phosphorus (%)	0.46
Salt	0.21	Metabolizable energy (Kcal/kg)	2,825
Soybean oil	2.21		
Total	100.00		

a Premix provided per kilogram of diet: 10,000 IU retinol; 1,800 IU cholecalciferol; 10 IU α-tocopherol; 10 mg menadione; 2.5 mg thiamine; 5.5 mg riboflavin; 3,000 mg nicotinic acid; 18 mg pantothenic acid; 4 mg pyridoxine; 0.02 mg cyanocobalamin; 4 mg biotin; 50 mg folic acid; 110 mg Mn; 65 mg Zn; 60 mg Fe; 7.5 mg Cu; 1.1 mg I; 0.15 mg Se.

Broilers were raised in net-floor pens within a poultry house that was thoroughly disinfected prior to the trial. The ambient temperature was maintained between 32 and 35 °C in brooding stage and adjusted to 18–24 °C in other stage, including appropriate ventilation and a humidity of 55 to 65%. The photoperiod cycle was set for 23:1 h of light and dark in brooding stage and adjusted to 18:6 in other stage. Birds were fed twice daily (08:00 and 16:00) with *ad libitum* access to water. Fresh feed was prepared daily. Hygiene was maintained, and morbidity/mortality were zero.

### 2.2 Preparation of fermented passion fruit peel (FPFP)

Passion fruit peel was procured from a market in Guangxi province. Fresh passion fruit peels were collected, crushed to an approximate length 3 mm, and the moisture content was adjusted to 55–60% using corn meal and rice bran. The treated passion fruit peel fermented with complex probiotics [on a viable count basis: *Candida utilis* (CICC 33699T) ≥90 × 10^6^ CFU/g, *Bacillus subtilis* (CICC 10089) ≥ 26 × 10^6^ CFU/g, *Pediococcus acidilactici* (CICC 10146) ≥ 4 × 10^6^ CFU/g]. The FPFP was rapidly sealed in specialised fermentation bags and fermented at room temperature for 30 days. The nutritional composition of the FPFP is provided in Table 2.

**Table 2:** Nutritional level of fermented passion fruit peel (dry matter basis).

Nutrient	Content (%)
Dry matter	94.43
Crude protein	11.43
Ash	9.45
Starch	6.53
Ether extract	5.56
Crude fiber	32.15
Calcium	0.58
Phosphorus	0.34

### 2.3 Growth performance

Throughout the experiment, broilers were weighed, and feed intake was recorded weekly. The average daily gain (ADG), average daily feed intake (ADFI), and the feed-to-gain ratio (F/G) were calculated.

### 2.4 Intestinal morphology

At the conclusion of the 49-day feeding trial, three birds from each pen (a total of sixty) were humanely euthanised prior to tissue collection. To minimise suffering and in accordance with the AVMA Guidelines for the Euthanasia of Animals, each bird was first rendered unconscious by a percussive blow to the head, immediately followed by complete exsanguination via severance of the carotid arteries and jugular veins. No chemical anaesthetics or sedatives were administered prior to the procedure. Following euthanasia, the abdominal cavity was immediately opened, and the segments of the small intestine (duodenum, jejunum, and ileum) were carefully dissected for morphological assessment. Segments of the small intestine were sampled from midpoint of duodenum; intestine from the gizzard to pancreatic and bile ducts, jejunum; midway between the point of entry of the bile ducts and Meckel’s diverticulum, Ileum; 10 cm proximal to the ileo-cecal junction. The intestinal samples were evaluated for the villus height, crypt depth and the ratio of villus height to crypt depth (V/C). Segments with 1.5 cm length were gently flushed twice with physiological saline solution (1% NaCl) to remove intestinal contents and were fixed in 4% paraformaldehyde. The samples were processed for 24 h in a tissue processor with ethanol as dehydrant and were embedded in paraffin. Sections were made and stained with hematoxylin–eosin. Villus height (VH) and crypt depth (CD) were measured using an image analysis system (Media Cybemetics, Image-Pro Plus 6.0, USA) under an optical microscope (Nikon, Eclipse Ci-L, Japan), and the villus height to crypt depth ratio (V/C) was calculated. VH represents the measurement from the tip of the villus to the lowest point between each villus, whereas CD refers to the distance between the opening of the crypt and its base. VH and CD of 10 unbroken villi were measured and their ratio (V/C) was calculated.

For haematoxylin and eosin (HE) staining, small intestinal tissue segments (duodenum, jejunum, and ileum) were first maintained in 75% ethanol at room temperature for subsequent histological processing. The tissues were then trimmed into 3–4 mm slices and fixed in 10% neutral buffered formalin. Following fixation, samples were dehydrated through a graded ethanol series, cleared in xylene, and embedded in paraffin blocks. Sections of 5–7 μm thickness were obtained using a microtome, stained with a standard HE staining kit (Beijing Solarbio Science & Technology Co., Ltd., Beijing, China), and examined for morphological assessment (22).

### 2.5 16S rRNA sequencing and cecal microbiota analysis

Cecal digesta were aseptically collected from three birds per group (close to the group average weight) immediately after euthanasia and stored at −80 °C. Total genomic DNA was extracted using the E.Z.N.A.® soil DNA Kit (Omega Bio-tek). The quality of the DNA was determined using NanoDrop ND-2000 Spectrophotometer (Thermo Fisher Scientific, Wilmington, USA). The purified DNA was subsequently used to create a 16S rRNA gene library, using an existing protocol (23). Briefly, PCR amplification of the V3-V4 region of the 16S rRNA gene was conducted using primers 338F (5′-ACTCCTACGGGAGGCAGCAG-3′) and 806R (5′-GGACTACHVGGGTWTCTAAT-3′). Purified amplicons were used for library construction, and high-throughput paired-end sequencing was performed on an Illumina Nextseq2000 platform (Shanghai Majorbio Bio-pharm Technology Co., Ltd.). After quality control and assembly of raw data, the DADA2 pipeline (plugin in the Qiime2, version 2020.2) was used for denoising to obtain amplicon sequence variants (ASVs) and an abundance table. Based on ASVs, taxonomic annotation, and alpha and beta diversity analyses were performed (24–27).

### 2.6 Statistical analysis

Alpha diversity indices, including the Chao 1 and Shannon indices, were calculated using the mothur software.^1^ Differences in alpha diversity between groups were assessed using the Wilcoxon rank-sum test. Similarities in microbial community structure among samples were examined by principal coordinate analysis (PCoA) based on Bray–Curtis distances. Permutational multivariate analysis of variance (PERMANOVA) was applied to test whether the differences in microbial community structure between sample groups were statistically significant. Linear discriminant analysis effect size (LEfSe)^2^ was employed to identify bacterial taxa with significantly different abundances from phylum to genus level among different groups (LDA score > 2, *p* < 0.05).

All data were analyzed by one-way ANOVA using SAS software (version 9.0, SAS Institute Inc., Cary, NC, USA), and the normality and homogeneity of variance were checked before statistical analysis. Each replicate was defined as an experimental unit for the trial. Statistical differences among treatment groups were compared using Duncan’s multiple comparison test. The results are presented as the means and standard error of the mean (SEM), and differences were considered significant at *p <* 0.05 and highly significant at *p <* 0.01.

## 3 Results

### 3.1 Growth performance

Dietary inclusion of FPFP significantly affected the growth performance of broilers (Table 3). Compared to the CK, L5, and L15 groups, the L10 group showed significantly higher final body weight, total weight gain, and average daily gain (*p <* 0.05). Specifically, the final body weight of the L10 group was 8.23, 13.28, and 16.32% higher than that of the CK, L5, and L15 groups, respectively (*p <* 0.01). Correspondingly, the average daily gain increased by 28.84, 30.71, and 41.89%, respectively (*p* < 0.01). No significant differences were observed in total feed intake or ADFI among groups (*p >* 0.05). The F/G of L10 group was significantly lower than that of all other groups (*p <* 0.05), with a highly significant difference compared to the L15 group (*p* < 0.01). No morbidity or mortality occurred during the trial.

**Table 3:** Effect of FPFP on growth performance of broilers.

Factor	CK	L5	L10	L15	*p*-Value
Initial weight (g)	1065.00 ± 186.61	993.34 ± 16.07	990.00 ± 82.61	1008.33 ± 36.32	0.800
Terminal weight (g)	1853.33 ± 97.51^b^	1770.45 ± 63.82^b^	2005.83 ± 28.76^ac^	1724.44 ± 97.64^bd^	0.010
Total weight gain (g)	788.33 ± 120.03^b^	777.11 ± 72.65^b^	1015.83 ± 95.00^a^	716.11 ± 64.81^b^	0.017
Total feed intake (g)	2424.82 ± 285.64	2290.73 ± 221.52	2508.35 ± 249.11	2403.26 ± 105.91	0.709
F/G	3.10 ± 0.32^a^	2.96 ± 0.30^a^	2.47 ± 0.13^bd^	3.37 ± 0.16^ac^	0.012
ADFI (g)	49.48 ± 5.83	46.75 ± 4.52	51.19 ± 5.08	49.05 ± 2.16	0.709
ADG (g)	16.09 ± 2.45^b^	15.86 ± 1.48^b^	20.73 ± 1.94^ac^	14.61 ± 1.32^bd^	0.017

F/G, feed-to-gain ratio; ADFI, average daily feed intake; ADG, average daily gain.

^a,b^Means within the same row that have no common superscript are significantly different (*p < 0.05*).

^c,d^Means within the same row that have no common superscript are significantly different (*p < 0.01*).

CK: control group; L5: group receiving a diet with 5% FPFP; L10: group receiving a diet with 10% FPFP; L15: group receiving a diet with 15% FPFP.

FPFP, fermented passion fruit peel.

The effect of FPFP on weekly weight gain showed dynamic changes (Table 4). During the first week, no significant difference was observed between CK and L10 groups (*p >* 0.05). From the third week onwards, weight gains across groups were similar, with no significant difference among groups (*p >* 0.05), although the CK and L10 groups exhibited higher values. During the final weeks (weeks 6–7), there was no significant difference among the groups. Collectively, the 10% inclusion level yielded the optimal growth performance.

**Table 4:** Effect of FPFP on weekly weight gain of broilers (g).

Weeks	CK	L5	L10	L15	*p*-Value
1	116.67 ± 10.41	63.89 ± 12.51	116.67 ± 63.92	54.45 ± 28.1	0.124
2	61.67 ± 41.63	72.22 ± 2.55	79.44 ± 4.19	46.11 ± 34.57	0.504
3	168.33 ± 42.52	117.78 ± 22.20	162.50 ± 8.70	117.78 ± 31.37	0.115
4	111.67 ± 22.55	107.78 ± 32.72	160.56 ± 45.65	128.89 ± 12.62	0.222
5	115.00 ± 55.30	126.78 ± 73.95	185.37 ± 55.00	112.22 ± 95.08	0.549
6	91.67 ± 33.29	118.22 ± 42.41	171.29 ± 22.68	117.78 ± 50.48	0.160
7	123.33 ± 77.51	170.45 ± 3.29	140.00 ± 24.74	138.89 ± 26.16	0.612

CK: control group; L5: group receiving a diet with 5% FPFP; L10: group receiving a diet with 10% FPFP; L15: group receiving a diet with 15% FPFP.

FPFP, fermented passion fruit peel.

### 3.2 Intestinal morphology

**Figure 1:**
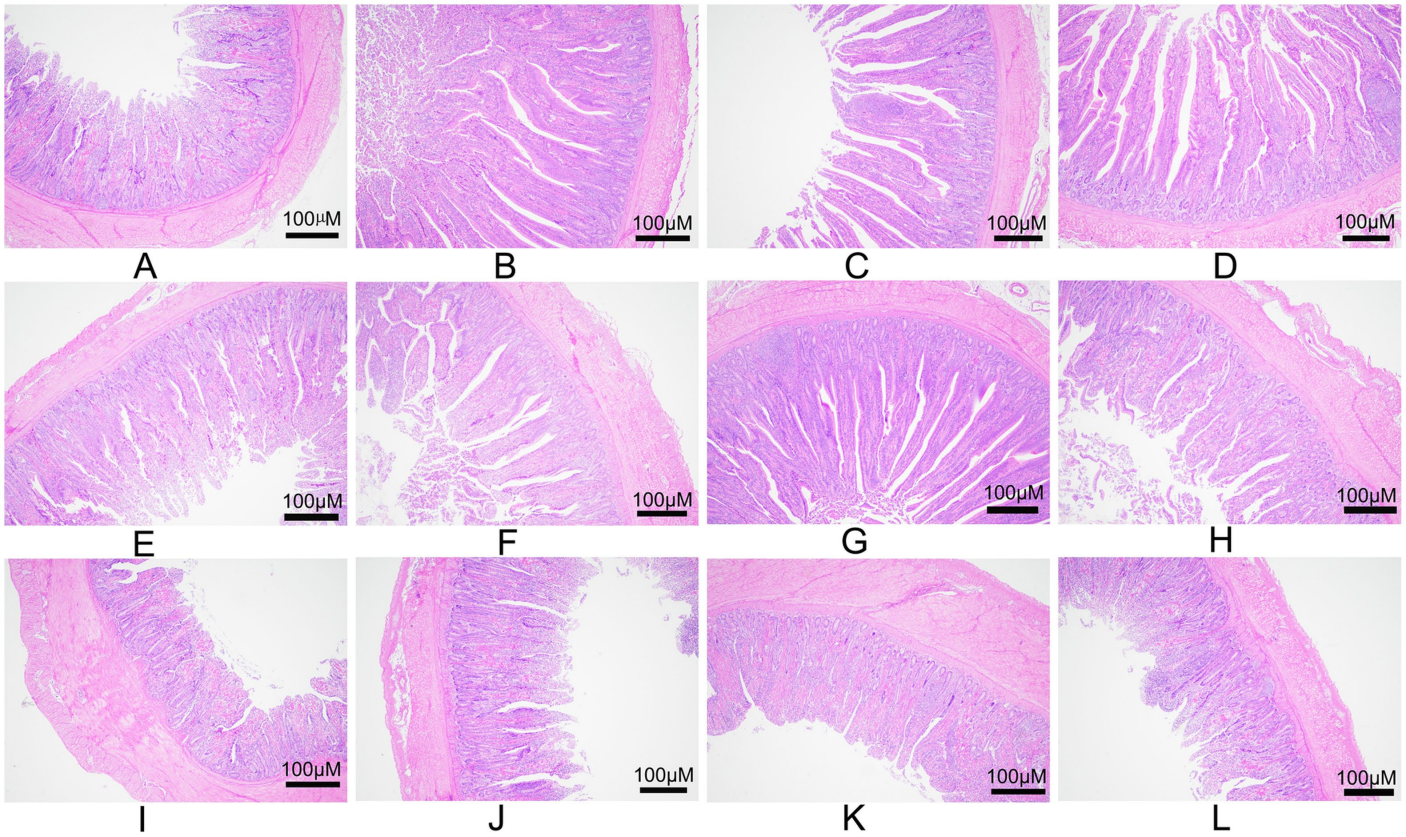
Morphology of the intestinal segments for the control and FPFP feeding of broilers. **(A–D)** Duodenum of the CK, L5, L10, and L15 groups; **(E–H)** Jejunum of the CK, L5, L10, and L15 groups; **(I–L)** Ileum of the CK, L5, L10, and L15 groups at 40 × magnification. Scale bar = 100 μm. CK: control group; L5: group receiving a diet with 5% FPFP; L10: group receiving a diet with 10% FPFP; L15: group receiving a diet with 15% FPFP. FPFP, fermented passion fruit peel.

The effect of dietary FPFP on intestinal morphology varied with the intestinal segment (Table 5, Figure 1). In duodenum, the villus height in L10 group was the highest, which was significantly higher than in the CK group (*p <* 0.05) but not significantly different from L5 and L15 groups. There was no significant difference in crypt depth and V/C among the treatment groups (*p >* 0.05). In jejunum, the villus height of CK and L10 group was significantly higher than that of L5 and L15 group (*p <* 0.05), but there was no significant difference between CK and L10 (*p >* 0.05). The depth of crypt in L10 group was the lowest crypt depth (through inter-group differences were not significant) and a V/C value of L10 group was significantly higher than that of the other groups (*p <* 0.05). In ileum, while villus height did not differ significantly among groups, crypt depth in L10 and CK groups was significantly lower than in L5 and L15 groups (*p <* 0.05). The V/C value of L10 group was also significantly higher than in the other three groups (*p <* 0.05).

**Table 5:** Effect of FPFP on intestinal morphology of broilers.

Index	CK	L5	L10	L15	*p*-Value
Villus height (μm, duodenum)	1044.34 ± 162.13	1380.03 ± 167.51	1170.43 ± 137.70	1159.11 ± 40.24	0.087
Villus height (μm, jejunum)	1268.20 ± 134.13^ac^	969.27 ± 2.76^bd^	1211.73 ± 52.04^ac^	978.13 ± 10.64^bd^	0.002
Villus height (μm, ileum)	577.97 ± 68.72	649.39 ± 193.44	611.10 ± 75.16	644.54 ± 24.34	0.843
Crypt depth (μm, duodenum)	344.46 ± 27.41	400.76 ± 71.63	329.55 ± 58.47	314.32 ± 38.10	0.267
Crypt depth (μm, jejunum)	367.54 ± 39.28	324.81 ± 62.39	275.51 ± 63.39	294.57 ± 95.46	0.425
Crypt depth (μm, ileum)	180.61 ± 31.73^b^	224.93 ± 8.70^ac^	148.56 ± 2.06^d^	224.07 ± 14.89^bc^	0.002
V/C (duodenum)	3.07 ± 0.73	3.50 ± 0.59	3.58 ± 0.32	3.73 ± 0.56	0.552
V/C (jejunum)	3.49 ± 0.61	3.06 ± 0.63	4.54 ± 0.92	3.60 ± 1.30	0.312
V/C (ileum)	3.23 ± 0.36	2.89 ± 0.89	4.11 ± 0.46	2.88 ± 0.08	0.067

V/C, villus height-to-crypt depth.

^a,b^Means within the same row that have no common superscript are significantly different (*p < 0.05*).

^c,d^Means within the same row that have no common superscript are significantly different (*p < 0.01*).

CK: control group; L5: group receiving a diet with 5% FPFP; L10: group receiving a diet with 10% FPFP; L15: group receiving a diet with 15% FPFP.

FPFP: fermented passion fruit peel.

### 3.3 Digestive organs development

Dietary inclusion of FPFP significantly increased the total intestinal length compared to the CK group (*p <* 0.05), with the value of L10 group showing the highest (Table 6). There was no significant difference in total intestinal weight and gizzard weight among groups (*p >* 0.05).

**Table 6:** Effects of FPFP on digestive system organs of broilers.

Factor	CK	L5	L10	L15	*p*-Value
Total length of intestine (cm)	134.67 ± 14.57^b^	171.00 ± 29.30^a^	180.33 ± 9.50^a^	171.33 ± 1.15^a^	0.046
Total intestinal weight (g)	96.20 ± 6.17	90.88 ± 16.54	96.23 ± 4.89	92.23 ± 20.07	0.120
Gizzard weight (g)	23.50 ± 2.26	25.27 ± 5.08	24.26 ± 6.16	29.17 ± 6.63	0.192

^a,b^Means within the same row that have no common superscript are significantly different (*p < 0.05*).

CK: control group; L5: group receiving a diet with 5% FPFP; L10: group receiving a diet with 10% FPFP; L15: group receiving a diet with 15% FPFP.

FPFP, fermented passion fruit peel.

### 3.4 Cecal microbiota

**Figure 2:**
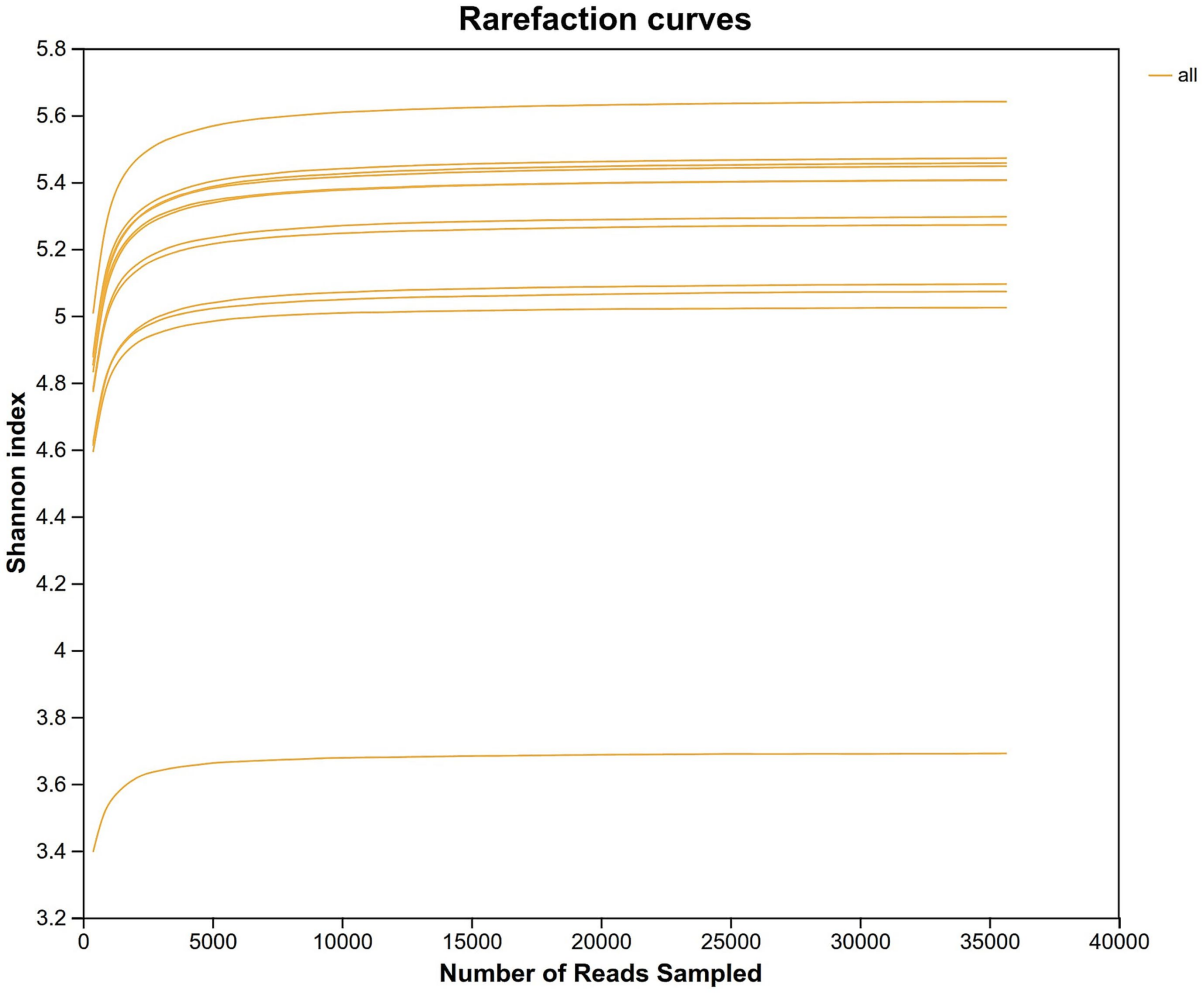
Dilution curve. The dilution curve is used to indicate whether the sequencing data volume of the sample is sufficient. When the curve tends to flatten towards the end, it indicates that the sequencing data volume is reasonable. The horizontal axis represents the volume of randomly selected sequencing data (number of reads sampled); the vertical axis represents the number of observed species or the alpha diversity index (Shannon index).

**Figure 3:**
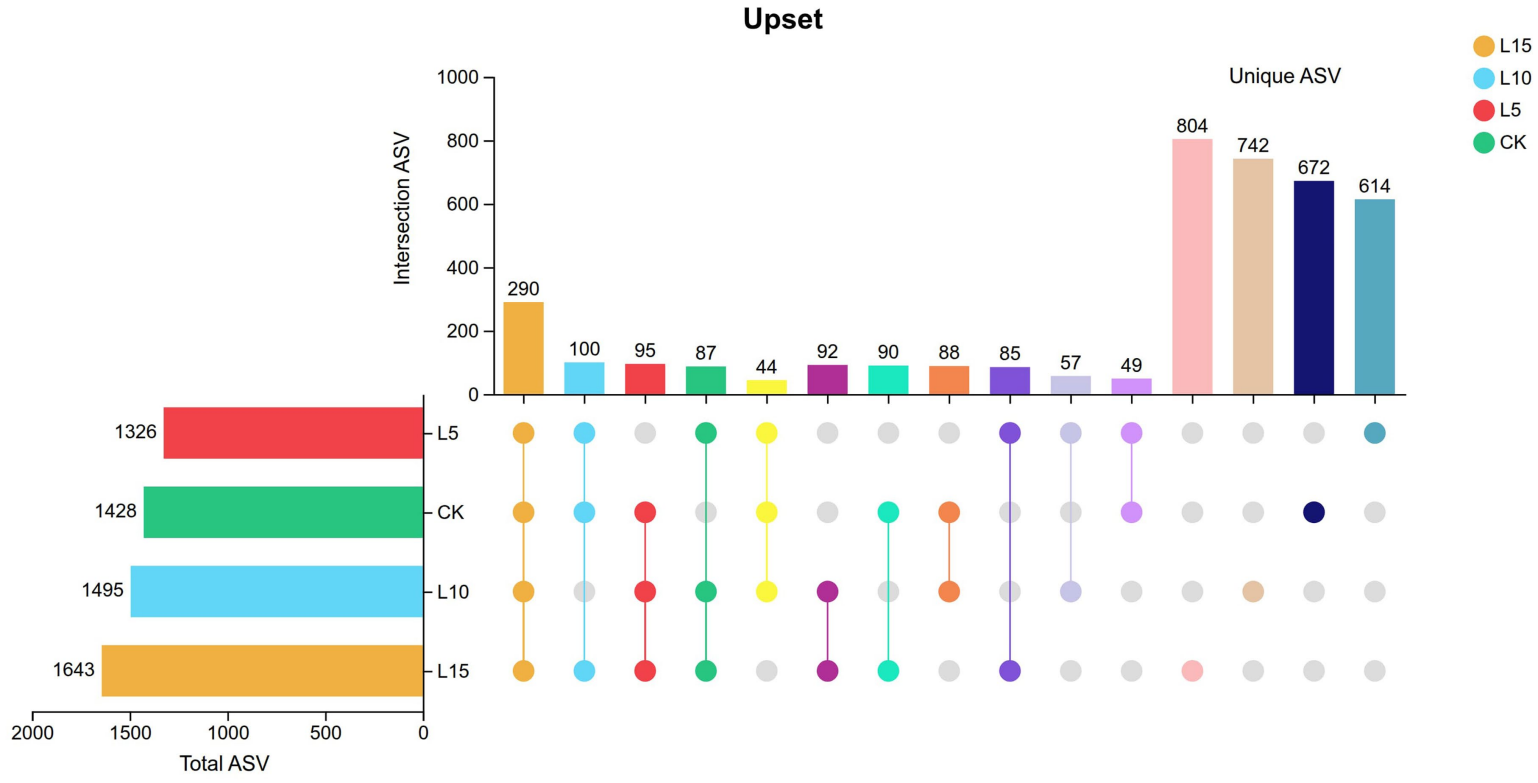
Effect of FPFP on cecal microbial community composition of broilers. The horizontal bar chart on the left represents the statistical values of elements for each sample group. In the central matrix, a single point denotes an element unique to a specific sample group, whilst connecting lines between points indicate the intersections of unique elements across different sample groups. The vertical bar charts on the right display the corresponding numerical values for these intersecting elements. CK: control group; L5: group receiving a diet with 5% FPFP; L10: group receiving a diet with 10% FPFP; L15: group receiving a diet with 15% FPFP. FPFP, fermented passion fruit peel.

**Figure 4:**
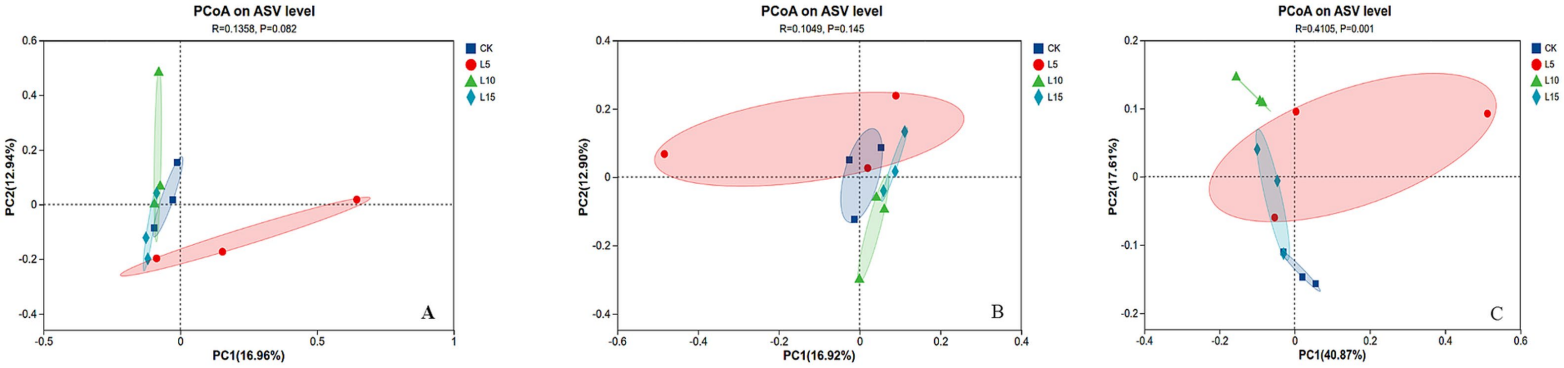
**(A–C)** Principal coordinate analysis of PCoA in the cecal microbial community of broilers. The horizontal and vertical axes correspond to the two selected principal coordinates (PC1 and PC2), with the percentages indicating the proportion of total variance in community composition explained by each coordinate. The axis scales represent unitless scores and are not directly interpretable as absolute distances. Data points are distinguished by color or shape according to their experimental group. The proximity between any two points reflects the similarity in their species composition. CK: control group; L5: group receiving a diet with 5% FPFP; L10: group receiving a diet with 10% FPFP; L15: group receiving a diet with 15% FPFP. FPFP, fermented passion fruit peel.

**Figure 5:**
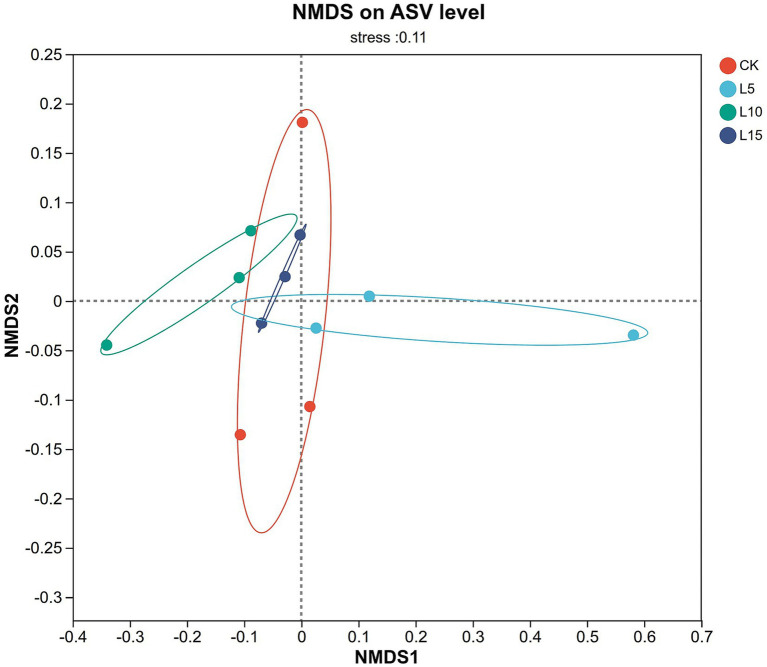
Non-metric multidimensional scaling analysis (NMDS). CK: control group; L5: group receiving a diet with 5% FPFP; L10: group receiving a diet with 10% FPFP; L15: group receiving a diet with 15% FPFP. FPFP, fermented passion fruit peel.

**Figure 6:**
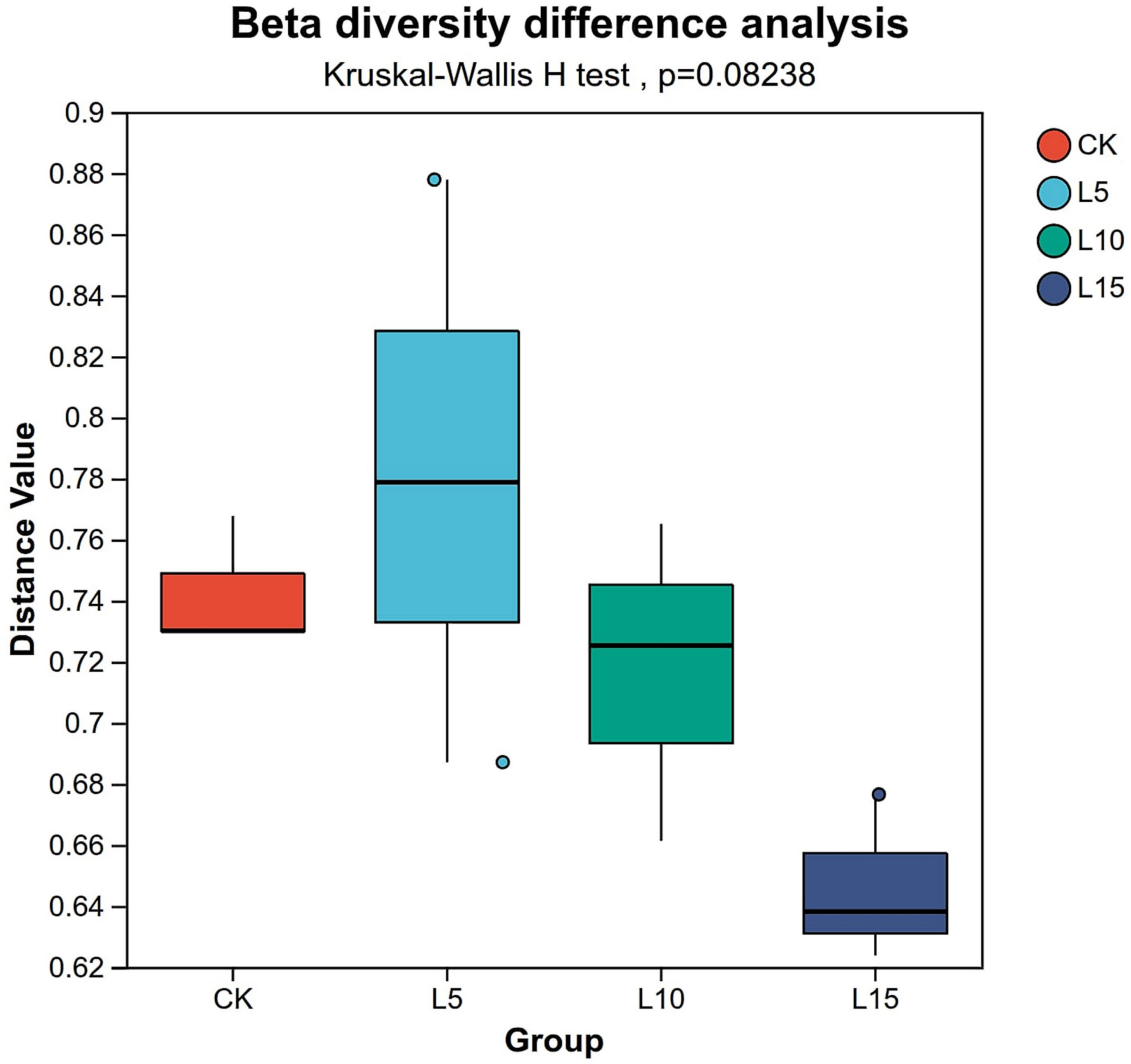
Inter group distance value analysis of beta diversity. The *N* box plots (where *N* equals the number of experimental groups) visualise the inter-group differences in beta diversity. Each box summarises the distribution of pairwise distances (e.g., Bray–Curtis or UniFrac) between all samples within its respective group. The dispersion of data within a single box (indicated by its interquartile range and whiskers) reflects the degree of compositional heterogeneity among samples in that group. The ordinate (*y*-axis) represents the calculated beta diversity distance value. CK: control group; L5: group receiving a diet with 5% FPFP; L10: group receiving a diet with 10% FPFP; L15: group receiving a diet with 15% FPFP. FPFP, fermented passion fruit peel.

**Figure 7:**
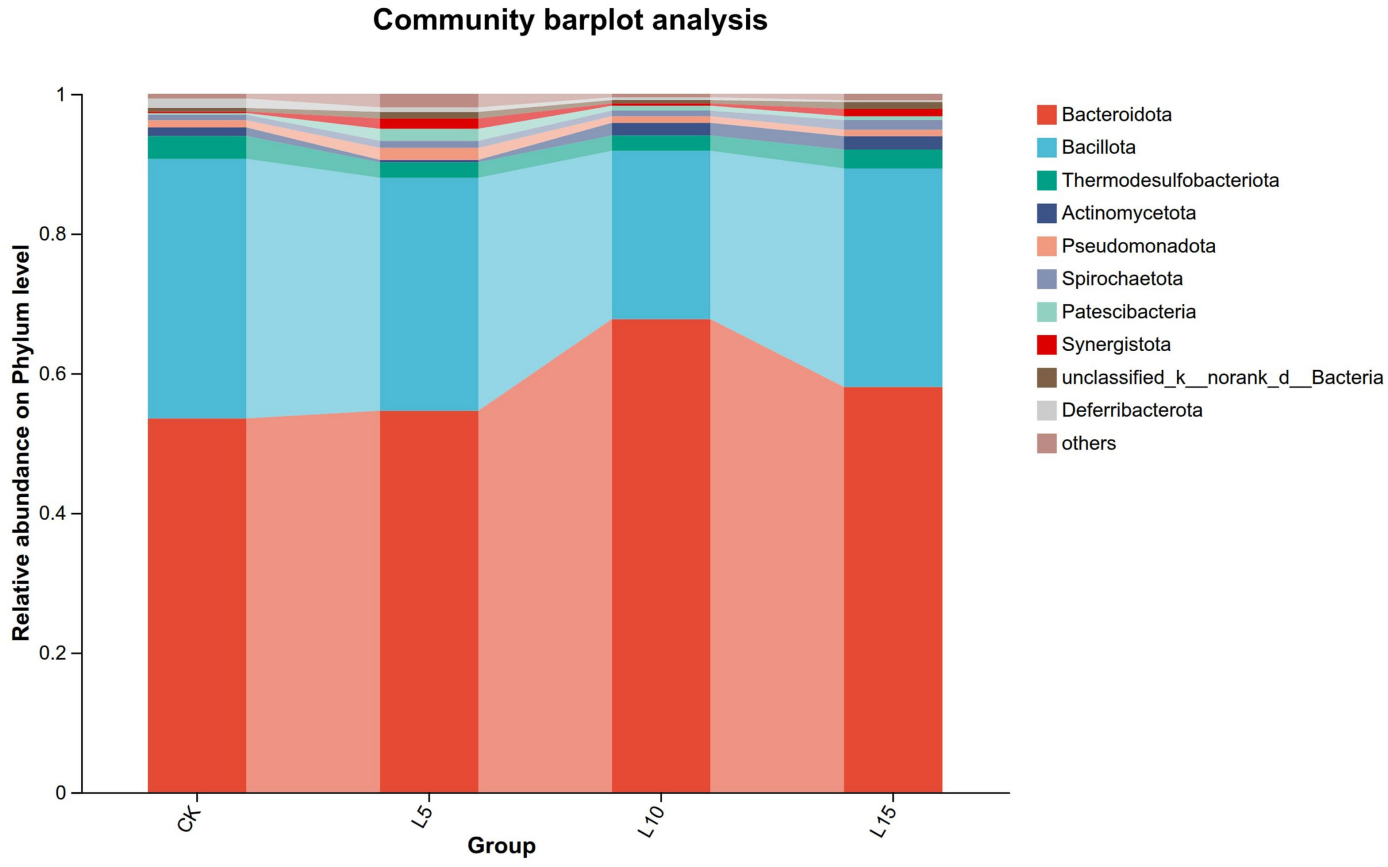
Relative abundance of cecal microbiota at phylum level. CK: control group; L5: group receiving a diet with 5% FPFP; L10: group receiving a diet with 10% FPFP; L15: group receiving a diet with 15% FPFP. FPFP, fermented passion fruit peel.

**Figure 8:**
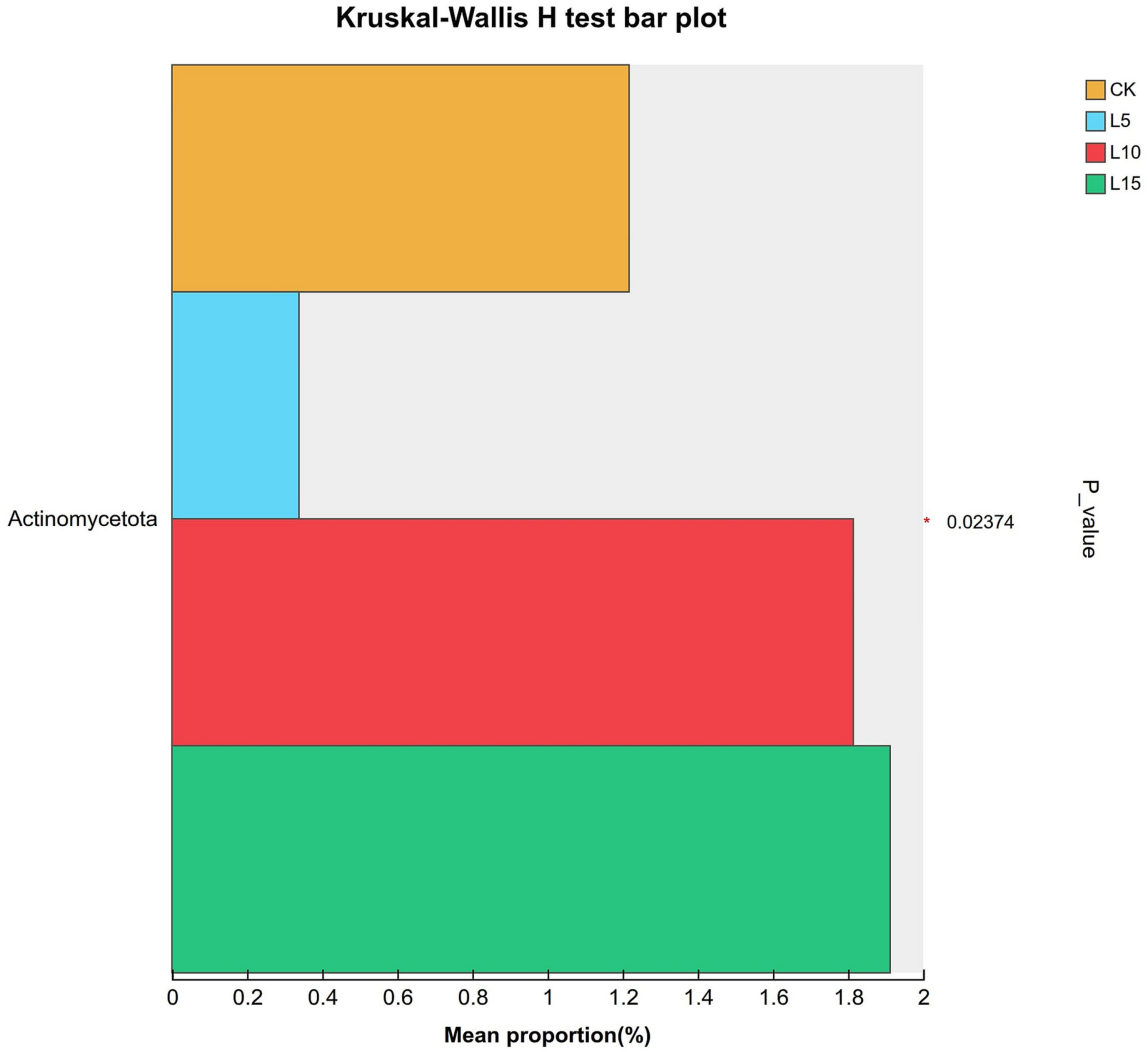
Kruskal–Wallis rank sum test among groups at phylum level. CK: control group; L5: group receiving a diet with 5% FPFP; L10: group receiving a diet with 10% FPFP; L15: group receiving a diet with 15% FPFP. FPFP, fermented passion fruit peel.

**Figure 9:**
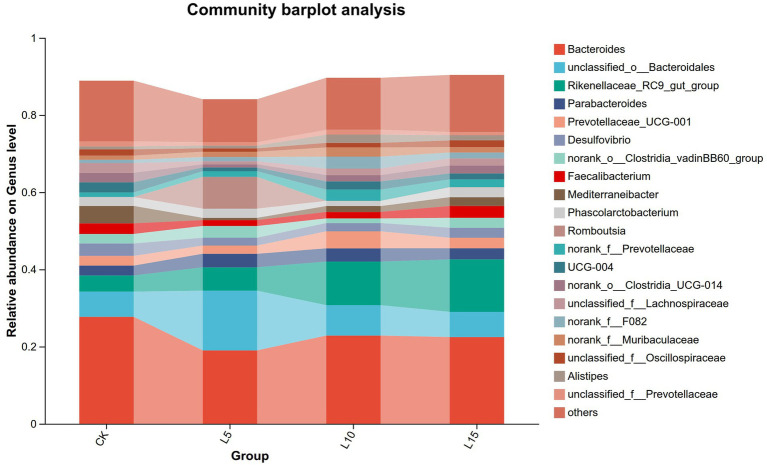
Relative abundance of cecal microbiota at genus level. CK: control group; L5: group receiving a diet with 5% FPFP; L10: group receiving a diet with 10% FPFP; L15: group receiving a diet with 15% FPFP. FPFP, fermented passion fruit peel.

**Figure 10:**
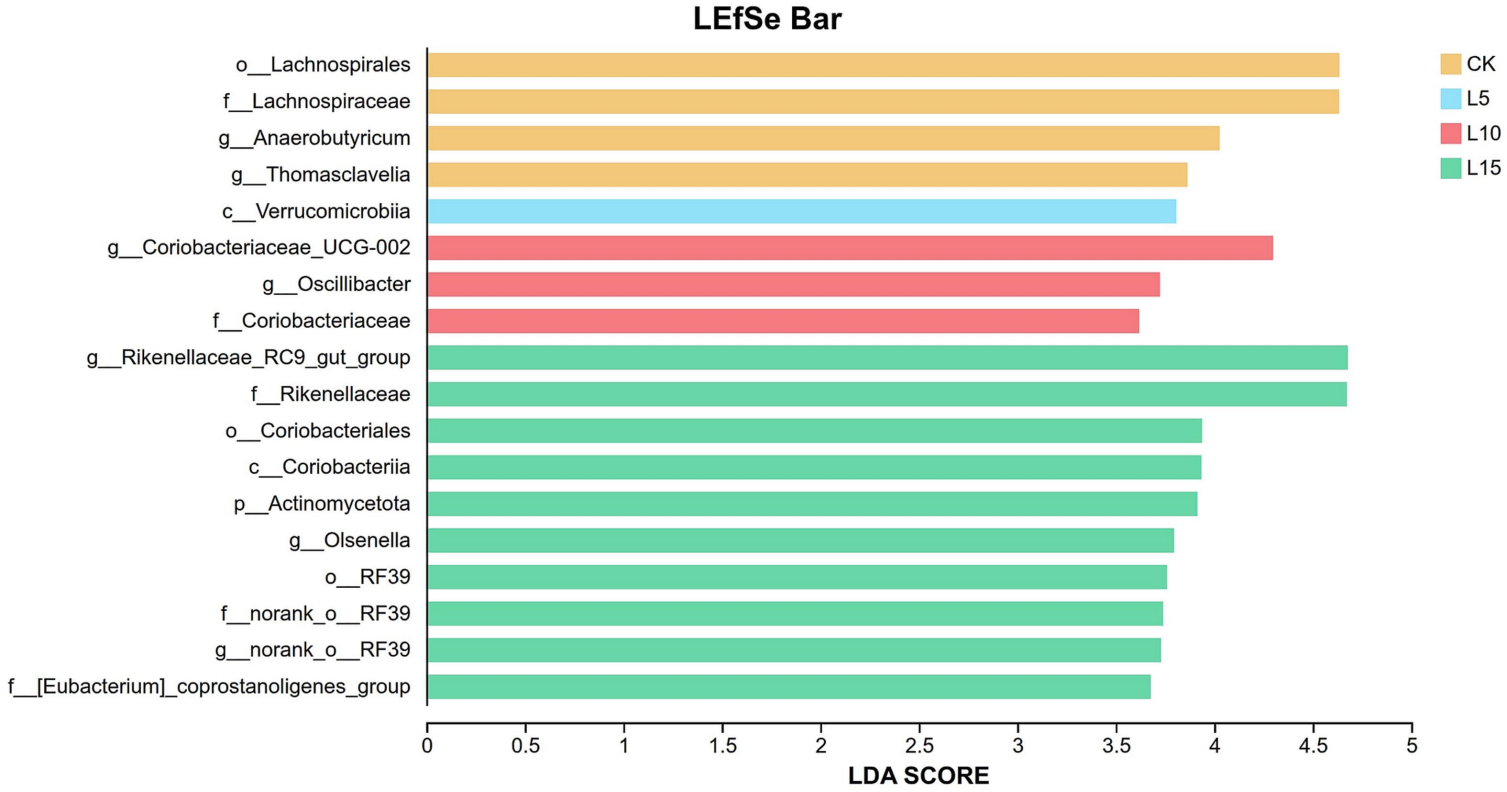
LDA effect size analysis (LEfSe). CK: control group; L5: group receiving a diet with 5% FPFP; L10: group receiving a diet with 10% FPFP; L15: group receiving a diet with 15% FPFP. FPFP, fermented passion fruit peel.

The ASV dilution curves for all groups plateaued with increasing sequencing depth (Figure 2), indicating sufficient coverage for reliable community analysis. The number of detected ASVs increased with higher FPFP inclusion: L5 (1326), CK (1428), L10 (1495), and L15 (1643) (Figure 3). A total of 290 ASVs were shared among all groups. Alpha diversity analysis (Table 7) showed that there was no significant difference among groups for the Chao1, ACE, Shannon, Simpson, and Sobs indices (*p > 0.05*), although the Chao1, ACE, and Sobs indices showed an increasing trend with higher FPFP inclusion. The Goods_coverage index was 1.00 for all samples. Principal coordinate analysis (PCoA) based on Bray–Curtis and Weighted UniFrac distances showed some visual separation (e.g., CK vs. L5) but no statistically significant inter-group differences according to PERMANOVA test (e.g., *R*^2^ = 0.1358, *p* = 0.082 for Bray–Curtis) (Figures 4A–C). Non-metric multidimensional scaling analysis (NMDS) and inter-group distance value analysis of beta diversity further confirmed no significant difference in microbial community structure among different treatment groups (Figures 5, 6). At the phylum level (Figure 7), the cecal microbiota of broilers was mainly dominated by Bacteroidota, Bacillota, Thermodesulfobacteriota, and Actinomycetota. The relative abundance of Bacteroidota was higher in treatment groups than in CK, though not significantly (*p > 0.05*, Figure 8). The relative abundance of Actinomycetota in group L5 was significantly lower than that in the other three groups (*p < 0.05*). At the genus level (Figure 9), the dominant bacteria included Bacteroides, unclassified o_Bacteroidales, Rikenellaceae_RC9_gut_group, and Parabacteroides. Differential analysis (Table 8) revealed that the abundances of Rikenellaceae_RC9_gut_group, *Olsenella*, and *Oscillibacter* were significantly higher in the L10 and L15 groups compared to the CK and L5 groups (*p < 0.05*). Conversely, the relative abundances of *Thomasclavelia* and *Anaerobutyricum* were significantly higher in the CK group (*p* < 0.05). LDA effect size analysis (LEfSe) analysis identified 18 biomarkers with significant difference abundances among the treatment groups (LDA score > 2.0, *p < 0.05*) (Figure 10). Biomarkers for the CK group included *Lachnospirales*, Lachnospiraceae, *Anaerobutyricum*, and *Thomasclavelia*. The biomarker of group L5 was Verrucomicrobiia. The biomarkers of group L10 included Coriobacteriaceae, Coriobacteriaceae_UCG-002, and *Oscillibacter*. L15 group had the most biomarkers, encompassing Actinomycetota, Coriobacteriia, Coriobacteriales, Rikenellaceae, Rikenellaceae_RC9_gut_group, *Olsenella*, norank o_RF39, and g_norank_o_RF39. These results showed that FPFP significantly regulated the composition and structure of intestinal microbiota in broilers.

**Table 7:** Alpha diversity analysis of cecal microorganisms.

Index	CK	L5	L10	L15	*p*-Value
ACE	641.07 ± 157.07	572.63 ± 247.28	662.14 ± 90.11	752.69 ± 33.80	0.668
Chao 1	635.89 ± 152.61	568.48 ± 243.26	655.88 ± 88.08	747.93 ± 35.72	0.668
Sobs	631.33 ± 147.14	559.33 ± 235.48	650.33 ± 84.42	739.00 ± 36.35	0.546
Shannon	5.25 ± 0.22	4.74 ± 0.93	5.26 ± 0.17	5.51 ± 0.12	0.270
Coverage	1.00	1.00	1.00	1.00	0.945
Simpson	0.01 ± 0.00	0.03 ± 0.04	0.01 ± 0.00	0.01 ± 0.00	0.459

CK: control group; L5: group receiving a diet with 5% FPFP; L10: group receiving a diet with 10% FPFP; L15: group receiving a diet with 15% FPFP.

FPFP, fermented passion fruit peel.

**Table 8:** Relative abundance of species with significant differences at genus level (%).

Genus name	Groups				*p*-Value
	CK	L5	L10	L15	
Rikenellaceae_RC9_gut_group	4.19 ± 1.26^b^	6.06 ± 2.68^b^	11.29 ± 3.94^a^	13.58 ± 3.68^a^	0.041
*Olsenella*	0.92 ± 0.18^a^	0.19 ± 0.12^b^	1.03 ± 0.06^a^	1.40 ± 0.33^a^	0.050
norank_o__RF39	1.10 ± 0.35^a^	0.25 ± 0.31^b^	0.31 ± 0.10^b^	1.21 ± 0.23^a^	0.038
norank_f__[*Eubacterium*]_coprostanoligenes_group	0.19 ± 0.15^b^	0.09 ± 0.05^b^	0.37 ± 0.12^a^	0.86 ± 0.50^a^	0.047
*Oscillibacter*	0.17 ± 0.04^b^	0.13 ± 0.10^b^	0.49 ± 0.38^a^	0.41 ± 0.15^a^	0.038
*Thomasclavelia*	0.95 ± 0.59^a^	0.04 ± 0.07^b^	0.01 ± 0.02^b^	0.16 ± 0.05^b^	0.029
*Solobacterium*	0.00 ± 0.00^b^	0.00 ± 0.00^b^	0.43 ± 0.07^a^	0.23 ± 0.20^a^	0.033
*Anaerobutyricum*	0.21 ± 0.09^a^	0.02 ± 0.03^b^	0.09 ± 0.01^b^	0.13 ± 0.02^b^	0.019
Coriobacteriaceae_UCG-002	0.02 ± 0.04^b^	0.00 ± 0.00^b^	0.09 ± 0.03^a^	0.02 ± 0.02^b^	0.031
norank__Puniceicoccaceae	0.01 ± 0.01^b^	0.06 ± 0.00^a^	0.00 ± 0.00^b^	0.00 ± 0.00^b^	0.022

^a,b^Means within the same row that have no common superscript are significantly different (*P < 0.05*).

CK: control group; L5: group receiving a diet with 5% FPFP; L10: group receiving a diet with 10% FPFP; L15: group receiving a diet with 15% FPFP.

FPFP, fermented passion fruit peel.

## 4 Discussion

Growth performance is a core economic indicator in poultry production (28). This study found that a 10% dietary inclusion of FPFP significantly enhanced final body weight, total gain, and ADG while reducing the F/G in Lingshan chickens, without affecting feed intake. The positive performance at the FPFP aligns with reports of improved growth from other fermented agro-byproducts like grape seed (29), sour cherry kernel (14) and plant product (30) in broiler diets. The growth and health of broilers are highly dependent on their diets, especially the diet rich in bioactive compounds (31). The bioactive compounds in passion fruit peel (1, 6), may contribute to this effect by enhancing immunity and antioxidant capacity (5, 9). The diet’s formulation and composition can potentially impact the growth performance and antioxidant capacity of animals, as stated in references (32–34). In this study, the 15% addition group had an unsatisfactory effect, which might be related to the excessively high crude fiber content in the diet, affecting the digestion and absorption of nutrients, suggesting the existence of an appropriate addition threshold.

The activity of animal digestive enzymes can be significantly influenced by the composition of the diet (35, 36). The health and function of the small intestine are reflected in villus architecture (37, 38). The significant increases in duodenal and jejunal villus height, along with the elevated V/C value in the jejunum and ileum observed in the 10% FPFP group, indicate improved digestive and absorptive surface area and function. These findings are consistent with studies demonstrating that appropriate dietary fibre from sources like ultrafine bamboo powder, soybean hulls, or wheat bran can enhance intestinal morphology in broilers (39–42). Dai et al. (39) reported a 28.77% increase in jejunal V/C value after feeding broilers a diet with 1% ultrafine bamboo powder fibre for 45 days; broilers fed 4% soybean hull fibre showed increased ileal villus height and maintained intestinal mucosal integrity (41); supplementing poultry diets with 4% sunflower seed hulls helped repair intestinal epithelial damage caused by bacterial infection (42); supplementing broiler diets with 3% wheat bran fibre benefited gizzard development, maintained intestinal morphological integrity, and increased digestive enzyme activity, thereby improving nutrient digestibility (40). Such fibres stimulate gizzard and intestinal development, prolong digesta retention, and promote enzyme secretion, thereby facilitating nutrient absorption (39, 43–45). The increased total intestinal length in FPFP-supplemented groups further supports the role of fibre in stimulating gastrointestinal tract development. However, excessive fibre can cause physical abrasion and reduce nutrient digestibility (44, 46), which may explain the morphological and growth performance limitations observed at the 15% inclusion level in this study.

An imbalance in the interplay between gut microbiota and other factors can disrupt the homeostasis of the intestinal mucosa (47). The intestinal epithelial barrier, acting as the first line of defense between the luminal environment and the host, can result in severe inflammation or other intestinal diseases if compromised (48, 49). The cecal microbiota plays a crucial role in host health and nutrient utilisation. In this study, while alpha and beta diversity metrics did not show significant structural shifts, notable changes occurred in specific taxa. The increasing trend in alpha diversity indices with FPFP inclusion suggests a potential for enhanced microbial abundance. Diversity indices provide an intuitive analysis of changes in microbial communities. A higher Chao1 index value indicates a greater number of species, and a higher Shannon index value and a lower Simpson index value signify higher microbial diversity within a sample (50, 51). Goods_coverage represents microbial coverage, and a value closer to 1 suggests that nearly all species in the sample were detected (51, 52). In this study, the coverage for all samples was close to 1.00, indicating the reliability of the data. ASV clustering analysis can accurately reflect species structure and diversity by differentiating microorganisms with 100% sequence similarity (53). Our results revealed a trend of increasing alpha diversity indices with higher inclusion levels, although no significant differences were observed among groups. Beta diversity analysis showed that the overall community structure was similar across groups, indicating that different inclusion levels did not cause drastic alterations in the cecal microbial community structure. At the phylum level, the dominance of Bacteroidota and Bacillota is typical in poultry (54). The higher relative abundance of Bacteroidota in treatment groups is relevant given its strong association with fibre degradation (55), suggesting improved capacity to utilise the fibrous components of FPFP. In this study, its relative abundance was higher in the treatment groups compared to the control group, and this correlated with the ADG, suggesting it may have facilitated the degradation and utilisation of crude fibre. Furthermore, Actinomycetota is implicated in immunomodulation and short-chain fatty acid production (56). Furthermore, the abundance of Actinomycetota was significantly lower in the 5% FPFP group compared to the other groups, while the 10 and 15% groups maintained relatively high levels. This may be related to the immunomodulatory effects of compounds such as flavonoids and polysaccharides present in passion fruit peel. At the genus level, the significant abundance of Rikenellaceae_RC9_gut_group [a known fibre-degrader; (57)] and *Oscillibacter* [linked to cholesterol metabolism and growth; (58, 59)] in the 10 and 15% groups is particularly compelling. These changes provide a mechanistic link between FPFP supplementation and positive growth performance. Therefore, the abundance of these bacteria likely enhanced the breakdown of dietary fibre and host metabolic efficiency. Furthermore, the distinct microbial biomarkers identified by LEfSe confirm that FPFP actively modulates the gut microbial community, even in the absence of wholesale community restructuring.

In our study, the observed increase in villus height could be partly attributed to the flavonoid content of FPFP, which has been shown in other studies to upregulate tight junction proteins in colonic tissue, alleviate intestinal inflammation, and promote intestinal barrier integrity (60). Furthermore, phenolic compounds have been demonstrated to significantly reverse the inflammation-induced increase in expression of MUC2 and TFF3 (61), which may be attributed to their ability to elevate intestinal secretory IgA (SIgA) levels (62) as well as other immunoglobulins (63). Polysaccharides can also reduce the expression of STAT3, NF-κB p65 and IκB in the intestinal epithelium, and inhibition of the STAT3/NF-κB pathway reverses the upregulation of inflammatory markers such as CHI3L1, IL-10 and IL-22, while increasing the expression of Reg-3β, Reg-3g and Reg-3γ and the proportion of goblet cells (64). Through these mechanisms, polysaccharides contribute to the repair of the damaged intestinal barrier, improve epithelial function, help restore gut microbiota balance, promote mucosal barrier integrity and alleviate ulcerative colitis.

## 5 Conclusion

This study demonstrates that dietary inclusion of 10% FPFP significantly enhances growth performance and improves feed efficiency in Guangxi Lingshan broilers. The beneficial effects are mediated through the following mechanisms. Firstly, the optimisation of intestinal morphology, characterised by increased villus height, higher villus height-to-crypt depth, and greater intestinal length. Secondly, the modulation of the cecal microbiota, specifically through the enrichment of key bacterial genera involved in fibre degradation (e.g., Rikenellaceae_RC9_gut_group) and host metabolism (e.g., *Oscillibacter*). These findings confirm the potential of FPFP as a valuable feed resource, contributing to sustainable poultry production.

## Data Availability

The data presented in the study are deposited in the National Center for Biotechnology Information (NCBI) Sequence Read Archive (SRA) repository, accession number PRJNA1442398.

## Footnotes

1
http://www.mothur.org/wiki/Calculators

2
http://huttenhower.sph.harvard.edu/LEfSe
